# Retroperitoneal necrotizing soft tissue infections: A case report and literature review

**DOI:** 10.1002/ccr3.6368

**Published:** 2022-09-20

**Authors:** Segni Kejela, Solomon Bekele

**Affiliations:** ^1^ Department of Surgery, College of Health Sciences Addis Ababa University Addis Ababa Ethiopia

**Keywords:** broad‐spectrum antibiotics, necrotizing soft tissue infection, retroperitoneal infections

## Abstract

Necrotizing soft tissue infections of the retroperitoneal is a rare disease identity. Here we present a 50‐year‐old male patient who underwent surgical exploration for retroperitoneal necrotizing soft tissue infection. Postoperatively, he was put on broad‐spectrum antibiotics. He passed on after the first exploration and debridement.

## Abstract 2

Retroperitoneal necrotizing soft tissue infections are rare but deadly entities. Maintain a high index of suspicion in patients with a typical peritoneal signs is mandatory for early detection.

## 1 INTRODUCTION

Necrotizing soft tissue infection is an entirely surgical diagnosis of friable necrotic fascia with dishwater exudate and absent purulent material in the anatomic region of interest.^1^ This is one of the most difficult diseases to diagnose and treat for both surgical and non‐surgical professionals.^2^ Its extreme rarity adds to the significant rate of misdiagnosis and delay in proper treatment of this infection further worsening the already grim prognosis.^3^, ^4^ Retroperitoneal necrotizing soft tissue infection is an extremely rare subgroup of necrotizing soft tissue infections with few case reports presented across the body of literature. Here, we present a case of a patient with retroperitoneal necrotizing soft tissue infection who underwent extensive debridement but died on the 3rd postoperative day. The aim is to review bodies of literature to find learning points and pitfalls on the management of the patient presented and help professional facing such cases acquire better insight.

## 2 CASE PRESENTATION

A 50‐year‐old male patient with no previously diagnosed comorbidities presented to our hospital with abdominal and bilateral flank pain of 6 days duration. The pain was ill‐defined in location from its onset and was dull in character with moderate severity. Subsequently, the pain intensity increased over the next 2 days and was associated with high‐grade fever, loss of appetite, and vomiting of ingested matter in multiple episodes. In association, he started to have complaints of diarrhea and right‐sided inguinal swelling which was noticed 5 days prior to his presentation to our hospital. He denied any history of alcohol use or smoking. He had no prior history of dyspepsia, weight loss, rectal bleeding, trauma, or diagnosis of renal stones.

On physical examination, the patient is sick looking in pain, with no sign of cardio‐respiratory distress, and well nourished. His vital signs were deranged with pulse rate of 110–116 beats/min, respiratory rate of 24 breaths/min, blood pressure of 140/80 mmHg, oxygen saturation of 88%–90%, and temperature of 37.3°C. Pertinent positive finding was found on abdominal examination which showed soft abdomen that moves with respiration and with tenderness all over the abdomen including bilateral flank areas. There was no sign of gross fluid collection in the peritoneum. There was a hard tender right inguinal fold swelling with overlying skin color change measuring 4 × 6 cm which was exquisitely tender. Perineal and digital rectal exam did not reveal any abnormal finding.

Complete blood count showed WBC of 10,700 cell/mm^3^, with neutrophil predominance of 92%. Hemoglobin was 14.7 g/dl, and platelet count was 163,000 cell/mm^3^. The renal and liver function tests were normal. Serum electrolyte showed potassium of 3.3 mEq/L. Urgent abdominal contrast CT scan was done which showed necrotic retroperitoneal right inguinal extensive necrosis extending from the pararenal area to the pararectal area with free air in the retroperitoneum. The patient was started on IV ceftriaxone and metronidazole, and resuscitated with 3 L of normal saline over 2 h and decision for surgical exploration was made.

**FIGURE 1:**
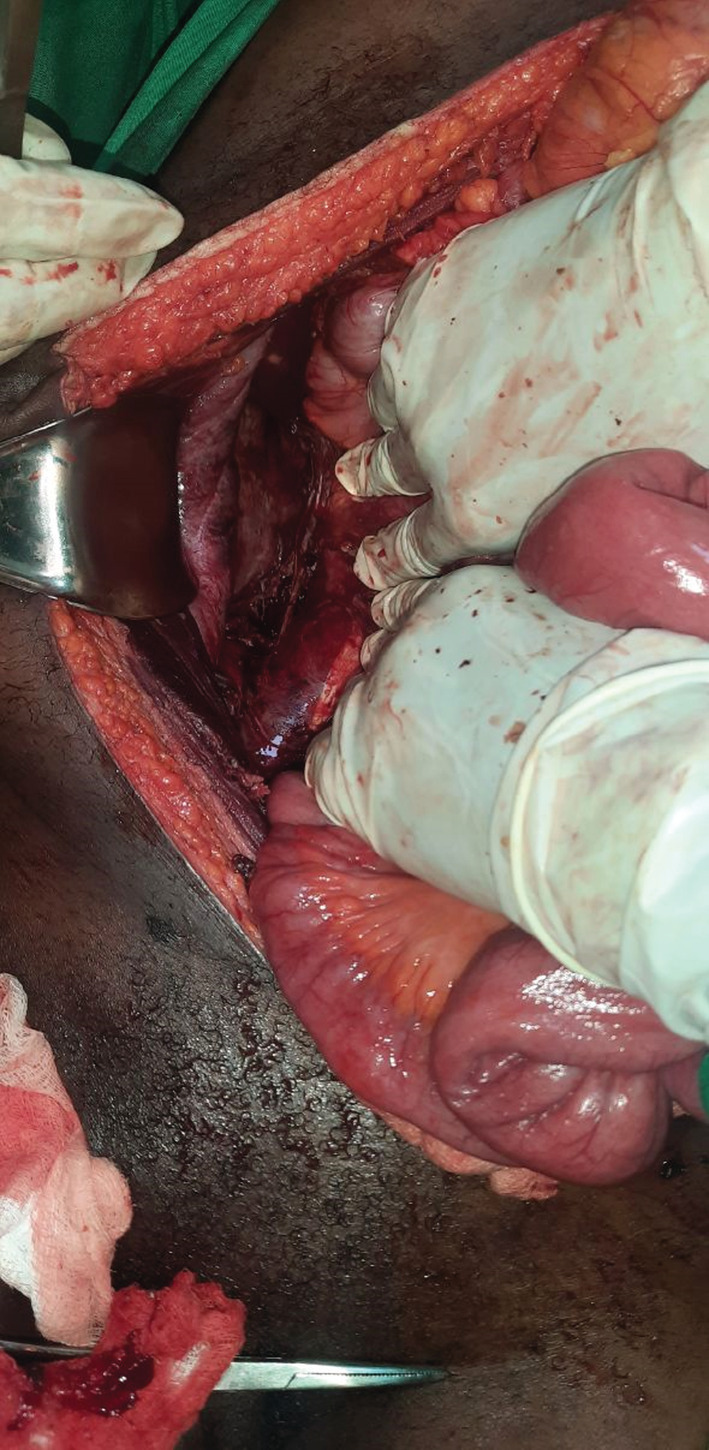
Right side retroperitoneum partly debrided off the necrotic tissues

**FIGURE 2:**
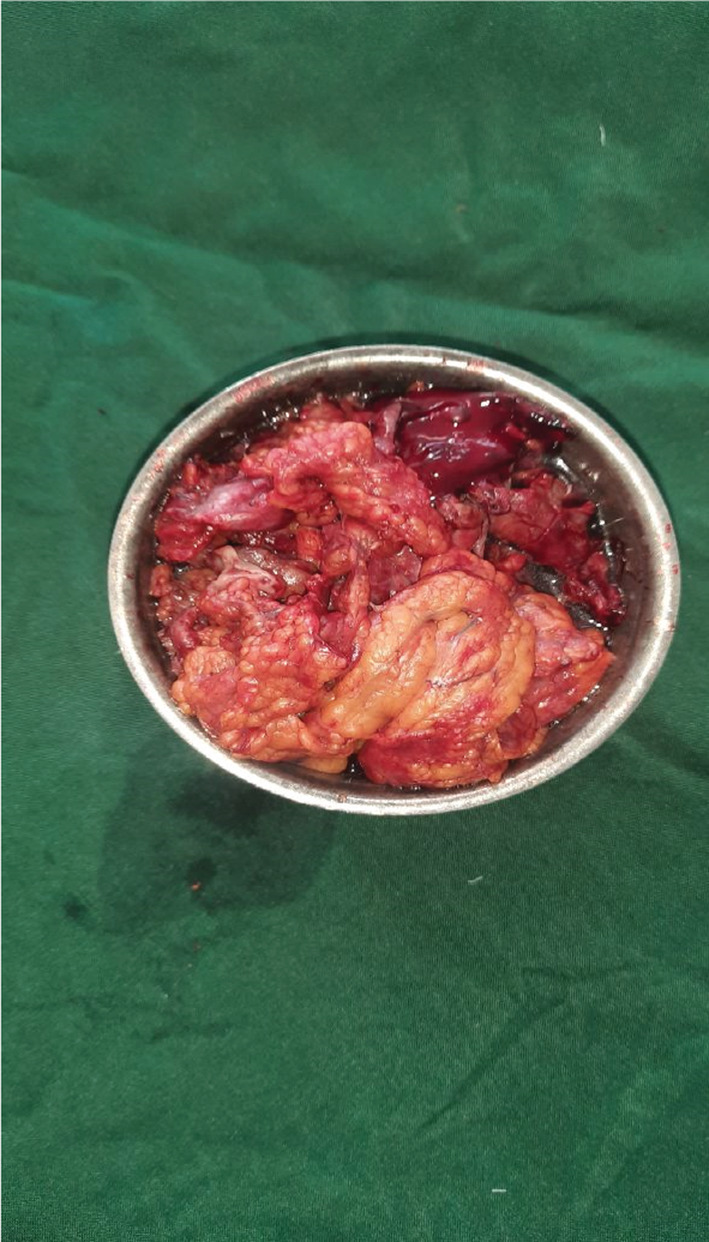
Debrided retroperitoneal fat transversalis fascia and peritoneum

The patient was taken to the operating theater, and under general anesthesia, exploratory laparotomy with a vertical mid‐line incision was made. Intraoperatively, 150 ml reactive fluid was found in the general peritoneum with no inflammatory sign in the peritoneal cavity. The retroperitoneum was accessed by dissecting anterior to the posterior rectus sheath and transversalis fascia, and necrotic retroperitoneal fibro‐fatty tissue with 900 ml foul‐smelling characteristic “dishwater” type content was found within the bilateral retroperitoneum. The kidneys, pancreas, large bowel, appendix, and rectum were grossly normal in appearance. All the necrotic tissue was debrided extensively including the peritoneum and transversalis fascia from pararenal area down to the pararectal area. (Figures 1 and 2) The right inguinal swelling was explored separately and necrotic inguinal lymph nodes and surrounding subcutaneous tissue not extending to the scrotum or right thigh. The area was also extensively debrided until viable tissue was identified circumferentially. Temporary abdominal closure was done with only skin closure with interrupted sutures for planned re‐debridement. Subsequently, the patient was continued on maintenance fluid, analgesics, and the antibiotics were changed to meropenem and vancomycin. Postoperatively, the patient's vital signs were pulse rate of 98 beats/min, respiratory rate of 24 breaths/min, blood pressure of 130/70 mmHg, and oxygen saturation of 94% with 3 L intranasal oxygen, with urine output of 0.8 ml/kg/min. Postoperative workup showed hemoglobin A1c of 7.2, and management for diabetes with modified sliding scale was initiated. Decision was made for surgical exploration at the 72nd hour of the postoperative course. But on the 70th postoperative hour, patient started to experience sudden onset shortness of breath and alteration in mentation. Upon reevaluation, the pulse rate was 120 beats/min which subsequently increased to 150 beats/min over 30 min and blood pressure of 80/40 mmHg. The respiratory rate decreased from 12 to 4 breath/minute with oxygen saturation of 40% on atmospheric air. The Glasgow Coma Scale reduced from 12 at initial evaluation after reported clinical deterioration to 4/15. ICU transfer was requested but was not available. Bag‐valve mask ventilation was started with fluid resuscitation, and subsequently, vasopressors were initiated, but 1 h after initiation of resuscitation the patient had cardio‐respiratory arrest, and CPR was unsuccessful, and patient passed on with possible cause of death of respiratory failure secondary to diaphragmatic paralysis secondary to extensive retroperitoneal necrotizing soft tissue infection.

## 3 DISCUSSIONS

Incidence of necrotizing soft tissue infection varies widely across studies from 0.3 cases per 100,000 to 15 cases per 100,000 population.^5^, ^6^, ^7^ Obesity, diabetes, immunocompromisation, alcohol use, and peripheral vascular diseases have all been cited as risk factors for necrotizing soft tissue infections.^2^ But all the risk factors sited are conclusions gathered from case reports, case series, and small retrospective studies.

Extensive literature review showed 14 cases of retroperitoneal necrotizing soft tissue infections from 13 case reports and one case series.^8^, ^9^, ^10^, ^11^, ^12^, ^13^, ^14^, ^15^, ^16^, ^17^, ^18^, ^19^, ^20^ The age of the patients ranged from 21 to 67 years with mean age of 48 years of age. Males were predominant with 11 of the 14 reported cases. Fournier's gangrene was the most common cause identified. Colonic pathologies were also diagnosed in three patients, and two of the patients had perforated colonic diverticula. One recent patient had COVID‐19 as a reported cause for the retroperitoneal necrotizing soft tissue infection. Nine of the 14 patients reported had no risk factor mentioned. Of the patients reported with risk factors, two patients had alcohol use disorder, tobacco chewing, diabetes, and HIV in one patient each. Twelve of the 14 patients we have gathered from the publications reported CT scan as a definitive diagnostic imaging, while two patients had intraoperative diagnosis. Six of the case reports detailed the antibiotics utilized. Meropenem, clindamycin, and 3rd‐ and 4th‐generation cephalosporines are more commonly utilized. All patients underwent exploration and extensive debridement. Eight of the 14 patients underwent single debridement while the rest underwent repeated debridement. Nine of the 14 patients were admitted to the ICU. Of the 12 patients who had microbial culture done, *Escherichia coli* (*E. coli*) was the most dominant bacterial etiology followed by different colonic origin anaerobes. Regarding the mortality, four of the 14 patients died while the rest were able to be discharged to home or rehabilitation centers. The details of the cases are elucidated in Table 1.^8^, ^9^, ^10^, ^11^, ^12^, ^13^, ^14^, ^15^, ^16^, ^17^, ^18^, ^19^, ^20^ It is worth noting that six of the patients reported having abscess drainage along with debridement for necrotizing soft tissue infection, so may not have strictly fulfilled the diagnostic criteria. In addition, the low mortality rate we have gathered may have been a subject of bias in publication of successfully managed cases only by authors and not a reflection of a real‐world mortality rate of the condition.

**TABLE 1:** Literature review of retroperitoneal necrotizing soft tissue infections

Age	Sex	Cause	Risk factors	Imaging	Antibiotic types	Number of debridement	Admission	Isolated organism	Outcome	Reference
52	M	Covid 19	None	CT	Not mentioned	1	ICU	*Klebsiella*, *E. coli*, anaerobes	Survived	Elashry et al.^8^
33	M	Trauma	Tobacco, Alcohol	CXR	Cefoperazone, Sulbactam, Clindamycin	1		*E. coli*	Survived	Giri et al.^9^
21	M	Gluteal abscess	None	CT	Ampicillin/sulbactam, Metronidazole, Amikacin	>3		*E. coli*	Survived	Agarwal et al.^10^
42	M	None	None	CT	Meropenem and Flagyl	1		*E. coli*, *Bacteroides fragilis*	Survived	Beg et al.^11^
35	M	Perianal abscess	None	CT	Not mentioned	1		*E. coli*, *Staphylococcus aureus*, *Pseudomonas*, *Acetinobacter lwoffi*	Survived	Anandhi et al.^12^
45	M	None	Alcohol	US, CT	Not mentioned	1	ICU	*Klebsiella*, *E. coli*	Dead	Anandhi et al.^12^
50	F	Colonic ovarian fistula	None	CT	Vancomycin, Cefepime, Clindamycin, Doxycycline	2	ICU	*E. coli*, *Bacteroides fragilis*, *Proteus mirabilis*	Dead	Gupta et al.^13^
50	M	Fournier's gangrene	DM	CT	Norvancomycin, Meropenem	>3		ESBL‐producing *E. coli*	Survived	He et al.^14^
43	F	Fournier's gangrene	None	None	Not mentioned	2	ICU	Not done	Survived	Abebe et al.^15^
62	M	Perforated diverticulitis	None	CT	Not mentioned	3	ICU	*E. coli*, *Streptococcus constellatus*, *Streptococcus milleri*, Anaerobes	Survived	Ranasinghe et al.^16^
55	M	Fournier's gangrene	HIV	CT	Piperacillin/tazobactam, Linezolid	>3	ICU	*Parabacteroides distasonis*, *Prevotella melaninogenica*, *Fusobacterium nucleatum*	Survived	Weimer et al.^17^
67	M	Sigmoid cancer	None	CT	Not mentioned	3	ICU	*E. coli* + anaerobes	Survived	Takakura et al.^18^
64	F	Perforated Colonic diverticulitis	Hypertension	CT	Not mentioned	1	ICU	Not done	Dead	Secil et al.^19^
55	M	Caliceal stone with chronic pyelonephritis	None	CT	Not mentioned	1	ICU	*E. coli*	Dead	Ammari et al.^20^

No study had published predictors of mortality in retroperitoneal necrotizing soft tissue infection patients. The four deaths reported had a relatively older mean age, 53.5 years, equal male to female ratio, and three of the four patients had derangement in renal function test either at presentation or on the subsequent in‐hospital days. Three patients had abscess drainage, and one patient was hypertensive and the other was alcoholic.

Several potential pitfalls in management of our case can be mentioned. Delay in presentation was partly because of the referring center's delay in diagnosis. Further delay was noted during investigation with a CT scan which added 18 hours of further delay to definitive management. Postoperatively, lack of ICU had led the patient to be admitted to the general wards which may have led to sub‐optimal monitoring. Furthermore, when the respiratory failure was detected, possibility of intubation and mechanical ventilation was again prohibited by lack of ICU bed and ventilators. In addition, earlier re‐debridement may have abated the progression of the infection.

## 4 CONCLUSION

Retroperitoneal necrotizing soft tissue infections are one of the most frequently misdiagnosed and mistreated infectious diseases known in the medical world. Diligent examination and timely imaging can lead to early diagnosis and management. Early and radical debridement, aggressive fluid resuscitation, use of vasopressors, and broad‐spectrum antibiotics would be needed as a bundle in caring for these patients.

## AUTHOR CONTRIBUTIONS

Both authors have contributed to the manuscript data collection, manuscript writing, and editing.

## CONFLICT OF INTEREST

Both authors declare no competing interest of any kind pertaining to this publication.

## DATA AVAILABILITY STATTEMENT

Not available due to privacy policy.

## ETHICAL APPROVAL

Ethical approval was not required according to our institution's review board policy. Written informed consent was obtained from both the patient and the family for this report and images accompanying it.

## CONSENT

Consent for publication was acquired from the patient and the family in a written form and can be obtained from the corresponding author.
